# Supplementary material for: Analysis of PPARγ Signaling Activity in Psoriasis
Source: Int J Mol Sci. 2021 Aug 10;22(16):8603. doi: 10.3390/ijms22168603 (PMC8395241; doi:10.3390/ijms22168603)
Supplement: Supplementary file 1 [file ijms-22-08603-s001.zip › Supplemental materials_Analysis of PPARg signaling activity in psoriasis/Pathway models/Models images and html files/Differentiation of psoriatic T cells/6317.html]

IL1RAP


# Protein IL1RAP

|  |  |
| --- | --- |
| URN | urn:agi-llid:3556 |
| Total Entities | 0 |
| Connectivity | 402 |
| Name | IL1RAP |
| Description | interleukin 1 receptor accessory protein |
| Notes | This gene encodes a component of the interleukin 1 receptor complex, which initiates signalling events that result in the activation of interleukin 1-responsive genes. Alternative splicing of this gene results in membrane-bound and soluble isoforms differing in their C-terminus. The ratio of soluble to membrane-bound forms increases during acute-phase induction or stress. [provided by RefSeq, Jul 2018] |
| Primary Cell Localization | Plasma membrane |
| Class | Receptor |

---

|  |  |
| --- | --- |
| Pathway | Osteoarthritis |
|  | Th17-Cell Differentiation |
|  | Bradykinin Effects in Inflammation |
|  | Toll-like Receptor Independent Sterile Inflammation |
|  | Mast-Cell Activation without Degranulation |
|  | Negative Acute Phase Proteins Synthesis |
|  | Positive Acute Phase Proteins Synthesis |
|  | Vascular Endothelial Cell Activation by Cytokines |
|  | IL1B Expression Targets -> Nociception |
|  | Lymphocyte Mediated Myocardial Injury in Myocarditis |
|  | Inflammatory Reaction in Acne Vulgaris |
|  | Keratinocyte Proliferation in Acne Vulgaris |
|  | T-Cells Differentiation Block in Psoriasis |
|  | Th17-Cell Function in Systemic Lupus Erythematosus |
|  | Vitiligo |
|  | T-Cell Cytotoxic Response against Melanocytes in Vitiligo |
|  | beta-Cell Destruction through Cytokines in Diabetes Mellitus |
|  | CD4+T-Cell Response in Celiac Disease |
|  | Leukocyte Migration toward the Endothelial Cell in Ulcerative Colitis |
|  | TSLP Signaling in Bronchial Epithelial Cell in Asthma |
|  | Th17-Cell Differentiation in Asthma |
|  | CFTR Expression in Epithelial Cells (Class I Mutations) |
|  | Osteoclast Activation in Gout |
|  | Neutrophil Recruitment in Sinovium in Gout |
|  | TNF and IL1B Induce Metalloproteinase Synthesis in Osteoarthritis |
|  | IL1B Induced Arthralgia |
|  | Th17-Cell and Th1 Immune Responsein Psoriatic Arthritis |
|  | Osteoclast Activation in Psoriatic Arthritis |
|  | Synovial Fibroblast Activation in Psoriatic Arthritis |
|  | Osteoclast Activation in Rheumatoid Arthritis |
|  | Synovial Fibroblast Activation by Citokines in Rheumatoid Arthritis |
|  | Th17-Cell Numbers Reduction in HIV |
|  | IL1 Receptor Antagonist (IL1RN) Deficiency (DIRA) |
|  | IL36 Receptor Antagonist (IL1F5) Deficiency (DITRA) |
|  | HMGB1 and IL1B in Neuroinflammation |
|  | HMGB1 and IL1B in Neuronal Hyperexcitation |
|  | Subepithelial Fibroblasts in Middle Ear Cholesteatoma |
|  | Cytokines Trigger Otitis Media |
|  | Mucin Hyperproduction in Goblet and Mucous Cells |
|  | Endothelial Cell Dysfunction in Glomerulonephritis |
|  | Endothelial Cell Dysfunction in Pyelonephritis |
|  | NFKB Canonical Signaling Activation in Cancer |
|  | STAT3 and NFkB Activate Inflammation-Induced Tumorigenesis |
|  | NFKB Signaling Activation by Blocking of Tumor Suppressors |
|  | IL1R -> STAT3 Signaling |
|  | IL1R -> NF-kB Signaling |
|  | NF-kB Canonical Signaling |
|  | IL1B Expression Targets |
|  | IL1A Expression Targets |
|  | PPAR Psoriasis |
|  | Model of PPARG signaling in psoriasis |
|  | 1\_Inflammation in keratinocytes in the pathology of acne |
|  | 1\_Differentiation of psoriatic T cells |
|  | 1\_TNF and IL1B provoke ECM degradation in osteoarthritis |
|  | 2\_Chondrocyte death in osteoarthritis |
|  | 2\_The role of endothelial cells in pyelonephritis |
|  | 1\_Insufficient activation of immune response in the middle ear epithelium cells in otitis media |
|  | 2\_Pathogens stimulate mucins expression in the middle ear |
|  | 3\_1\_Mucus accumulation in asthma |
|  | 4\_Mucin hyperproduction in goblet and mucous cells in COPD |
|  | 3\_Leukocyte migration toward the endothelial cells in the intestine microvasculature |
|  | 1\_CFTR expression and degradation in epithelial cells in cystic fibrosis (class I-II mutations) |
|  | 1\_Antigen-presenting cells promote islet dysfunction and immune system activation in T1D |
|  | drug-target moltransport |
|  | Differentiation of psoriatic T cells |

---

|  |  |
| --- | --- |
| MedScan ID | 3556 |

---

|  |  |
| --- | --- |
| LocusLink ID | 3556 |
|  | 16180 |
|  | 25466 |

---

|  |  |
| --- | --- |
| Alias | interleukin IL1R3 |
|  | 6430709H04Rik |
|  | IL-1RACP |
|  | OTTHUMP00000209490 |
|  | interleukin IL1 receptor accessory protein beta |
|  | interleukin IL-1RACP |
|  | OTTHUMP00000209488 |
|  | OTTHUMP00000209486 |
|  | interleukin IL1 receptor accessory protein |
|  | Il1racpb |
|  | interleukin-1 receptor 3 |
|  | FLJ37788 |
|  | MGC114349 |
|  | interleukin IL-1R AcP |
|  | AI255955 |
|  | AV239853 |
|  | interleukin IL1 RAP |
|  | OTTHUMP00000209492 |
|  | OTTHUMP00000209491 |
|  | IL-1R AcP |
|  | interleukin-1 receptor accessory protein beta |
|  | IL-1 receptor accessory protein |
|  | OTTHUMP00000209487 |
|  | C3orf13 |
|  | RIKEN cDNA 6430709H04 gene |
|  | interleukin 1 receptor accessory protein |
|  | IL1 RAP |
|  | IL1R3 |
|  | OTTHUMP00000209489 |
|  | interleukin-33 receptot beta chain |
|  | interleukin-1 receptor accessory protein |
|  | IL-1R3 |
|  | IL-1R-3 |

---

|  |  |
| --- | --- |
| GO ID | 0004908 |
|  | 0005149 |
|  | 0002114 |
|  | 0019221 |
|  | 0006955 |
|  | 0006954 |
|  | 0045087 |
|  | 0070498 |
|  | 0042094 |
|  | 0038172 |
|  | 0072602 |
|  | 0051092 |
|  | 0032736 |
|  | 0032754 |
|  | 2000778 |
|  | 0051965 |
|  | 0065003 |
|  | 0099151 |
|  | 1905606 |
|  | 0099560 |
|  | 0099545 |
|  | 0005576 |
|  | 0098978 |
|  | 0005887 |
|  | 0016020 |
|  | 0005886 |
|  | 1990782 |
|  | 0002376 |
|  | 1900006 |
|  | 0007165 |
|  | 0005737 |
|  | 0005615 |
|  | 0016021 |
|  | 0032991 |
|  | 0019966 |
|  | 0030674 |
|  | 0004871 |
|  | 0006461 |
|  | 0043234 |
|  | 0005622 |
|  | 0004888 |
|  | 0031224 |
|  | 0004872 |

---

|  |  |
| --- | --- |
| KEGG ID | rno:25466 |
|  | hsa:3556 |
|  | mmu:16180 |

---

|  |  |
| --- | --- |
| Organism | Homo sapiens {Organism urn:agi-taxid:9606} |
|  | Mus musculus {Organism urn:agi-taxid:10090} |
|  | Rattus norvegicus {Organism urn:agi-taxid:10116} |
|  | Mus musculus |
|  | Homo sapiens |
|  | Rattus norvegicus |

---

|  |  |
| --- | --- |
| Mouse chromosome position | 16 |

---

|  |  |
| --- | --- |
| OMIM ID | 602626 |

---

|  |  |
| --- | --- |
| Rat chromosome position | 11q22 |

---

|  |  |
| --- | --- |
| Hugo ID | 5995 |
|  | HGNC:5995 |

---

|  |  |
| --- | --- |
| Human chromosome position | 3q28 |

---

|  |  |
| --- | --- |
| Swiss-Prot Accession | A8K6K4 |
|  | Q9NPH3 |
|  | Q9NPH3.2 |
|  | E9Q6I2 |
|  | Q3V2X3 |
|  | Q61730 |
|  | Q3UEL3 |
|  | Q61730.1 |
|  | D1M8S3 |
|  | Q63621 |
|  | Q63621.1 |
|  | B1NLD0 |
|  | D3DNW0 |
|  | O14915 |
|  | Q86WJ7 |
|  | Q3UVZ1 |
|  | Q8VCB9 |
|  | Q8N9C1 |
|  | Q4V8K9 |
|  | Q76LI3 |

---

|  |  |
| --- | --- |
| PIR ID | A57535 |

---

|  |  |
| --- | --- |
| GenBank ID | NC\_000003 |
|  | NR\_157352 |
|  | NR\_157353 |
|  | NM\_001167931 |
|  | NP\_001161403 |
|  | NM\_001364879 |
|  | NP\_001351808 |
|  | NM\_001167928 |
|  | NP\_001161400 |
|  | NM\_001364881 |
|  | NP\_001351810 |
|  | NM\_002182 |
|  | NP\_002173 |
|  | NM\_001167929 |
|  | NP\_001161401 |
|  | NM\_001364880 |
|  | NP\_001351809 |
|  | NM\_134470 |
|  | NP\_608273 |
|  | NM\_001167930 |
|  | NP\_001161402 |
|  | XM\_017006348 |
|  | XP\_016861837 |
|  | NG\_029105 |
|  | AC008249 |
|  | AC108747 |
|  | AF016261 |
|  | AAC39609 |
|  | AH009309 |
|  | AAF71688 |
|  | AAF71689 |
|  | CH471052 |
|  | EAW78100 |
|  | EAW78101 |
|  | EAW78102 |
|  | EAW78103 |
|  | AB006537 |
|  | BAA25421 |
|  | AF029213 |
|  | AAB84059 |
|  | AF167343 |
|  | AAF71687 |
|  | AF487335 |
|  | AAO49451 |
|  | AF538730 |
|  | AAQ01755 |
|  | AF538731 |
|  | AAQ01756 |
|  | AF538732 |
|  | AAQ01757 |
|  | AF538733 |
|  | AAQ01758 |
|  | AF538734 |
|  | AAQ01759 |
|  | AK095107 |
|  | BAC04487 |
|  | AK291669 |
|  | BAF84358 |
|  | BC016141 |
|  | BC053621 |
|  | AAH53621 |
|  | BG032519 |
|  | BM194193 |
|  | BM760155 |
|  | BM973472 |
|  | DA024801 |
|  | DN990628 |
|  | EF591790 |
|  | ABU90811 |
|  | FJ998418 |
|  | ACR82488 |
|  | HY121126 |
|  | Q9NPH3 |
|  | NC\_000082 |
|  | XR\_875736 |
|  | XR\_384528 |
|  | NM\_001159318 |
|  | NP\_001152790 |
|  | NM\_001159317 |
|  | NP\_001152789 |
|  | NM\_008364 |
|  | NP\_032390 |
|  | NM\_134103 |
|  | NP\_598864 |
|  | NR\_152416 |
|  | AC154234 |
|  | AC154601 |
|  | CH466521 |
|  | EDK97690 |
|  | EDK97691 |
|  | EDK97692 |
|  | CT009561 |
|  | AK032620 |
|  | AK033972 |
|  | AK034269 |
|  | AK039582 |
|  | BAC30392 |
|  | AK045686 |
|  | BAC32457 |
|  | AK083209 |
|  | BAE43382 |
|  | AK136782 |
|  | BAE23128 |
|  | AK149467 |
|  | BAE28898 |
|  | BC021159 |
|  | AAH21159 |
|  | BU526001 |
|  | X85999 |
|  | CAA59991 |
|  | Q61730 |
|  | NC\_005110 |
|  | XR\_001840406 |
|  | XM\_006248504 |
|  | XP\_006248566 |
|  | NM\_001167840 |
|  | NP\_001161312 |
|  | NM\_012968 |
|  | NP\_037100 |
|  | AC\_000079 |
|  | AABR07072255 |
|  | AAHX01070037 |
|  | AAHX01070038 |
|  | CH473999 |
|  | EDL78120 |
|  | AB079119 |
|  | BAD15354 |
|  | BC097339 |
|  | AAH97339 |
|  | GU123169 |
|  | ACY68959 |
|  | U48592 |
|  | AAB03502 |
|  | Q63621 |
|  | XP\_024309279 |
|  | XM\_017006347 |
|  | XP\_016861836 |
|  | XM\_024453511 |
|  | AF167333 |
|  | AF167334 |
|  | XM\_005247433 |
|  | XP\_005247490 |
|  | XM\_011512794 |
|  | XP\_011511096 |
|  | XM\_005247434 |
|  | XP\_005247491 |
|  | NC\_018914 |
|  | AF167335 |
|  | AF167336 |
|  | AF167337 |
|  | AF167338 |
|  | AF167339 |
|  | AF167340 |
|  | AF167341 |
|  | AF167342 |
|  | AMYH02008408 |
|  | AMYH02008409 |
|  | HQ448120 |
|  | ADQ32601 |
|  | AC\_000038 |
|  | AAHY01126468 |
|  | AAHY01126469 |
|  | AAHY01126470 |
|  | AC\_000135 |
|  | ABBA01058325 |
|  | ABBA01058326 |
|  | ABBA01058327 |
|  | ABBA01058328 |
|  | NT\_005612 |
|  | NW\_001838884 |
|  | NW\_921807 |
|  | AC\_000046 |
|  | CR591396 |
|  | Q8N9C1 |
|  | NT\_039624 |
|  | NW\_001030584 |
|  | Q3UEL3 |
|  | Q3UVZ1 |
|  | Q3V2X3 |
|  | NW\_047356 |
|  | NW\_001084663 |
|  | Q4V8K9 |
|  | Q76LI3 |

---

|  |  |
| --- | --- |
| Swiss-Prot ID | IL1AP\_RAT |
|  | IL1AP\_HUMAN |
|  | IL1AP\_MOUSE |
|  | D1M8S3\_RAT |
|  | A8K6K4\_HUMAN |
|  | Q3UEL3\_MOUSE |
|  | E9Q6I2\_MOUSE |
|  | Q3V2X3\_MOUSE |
|  | Q3UVZ1\_MOUSE |

---

|  |  |
| --- | --- |
| Cell Localization | Membrane |
|  | Cell membrane |
|  | Secreted |
|  | Plasma membrane |

---

|  |  |
| --- | --- |
| Ensembl ID | ENSG00000196083 |
|  | ENSP00000401132.1 |
|  | ENST00000439062.6 |
|  | ENSP00000072516.3 |
|  | ENST00000072516.7 |
|  | ENSP00000387371.1 |
|  | ENST00000422940.5 |
|  | ENSP00000314807.3 |
|  | ENST00000317757.7 |
|  | ENSP00000390541.1 |
|  | ENST00000447382.6 |
|  | ENSP00000409352.1 |
|  | ENST00000422485.5 |
|  | ENSMUSG00000022514 |
|  | ENSMUSP00000128100.2 |
|  | ENSMUST00000166294.8 |
|  | ENSMUSP00000093843.2 |
|  | ENSMUST00000096129.8 |
|  | ENSMUSP00000023156.6 |
|  | ENSMUST00000023156.12 |
|  | ENSMUSP00000134202.1 |
|  | ENSMUST00000174202.7 |
|  | ENSRNOG00000001928 |
|  | ENSRNOP00000051260.4 |
|  | ENSRNOT00000043498.6 |
|  | ENSRNOP00000002645.3 |
|  | ENSRNOT00000002645.4 |
|  | ENST00000447382.5 |
|  | ENST00000439062.5 |
|  | ENSP00000401132 |
|  | ENST00000439062 |
|  | ENSP00000072516 |
|  | ENST00000072516 |
|  | ENSP00000387371 |
|  | ENST00000422940 |
|  | ENSP00000314807 |
|  | ENST00000317757 |
|  | ENSP00000390541 |
|  | ENST00000447382 |
|  | ENSP00000409352 |
|  | ENST00000422485 |
|  | ENSP00000408893 |
|  | ENST00000443369 |
|  | ENSMUSP00000128100 |
|  | ENSMUST00000166294 |
|  | ENSMUSP00000093843 |
|  | ENSMUST00000096129 |
|  | ENSMUSP00000023156 |
|  | ENSMUST00000023156 |
|  | ENSMUSP00000134202 |
|  | ENSMUST00000174202 |
|  | ENSRNOP00000051260 |
|  | ENSRNOT00000043498 |
|  | ENSRNOP00000002645 |
|  | ENSRNOT00000002645 |

---

|  |  |
| --- | --- |
| MGI ID | MGI:104975 |
|  | 104975 |

---

|  |  |
| --- | --- |
| RGD ID | 2893 |

---

|  |  |
| --- | --- |
| Unigene ID | Rn.10511 |
|  | Hs.478673 |
|  | Mm.253424 |

---

|  |  |
| --- | --- |
| Homologene ID | 1643 |

---

|  |  |
| --- | --- |
| Shape | Stick-vertex |

---

|  |  |
| --- | --- |
| IPI ID | IPI00031789 |
|  | IPI00107619 |
|  | IPI00337687 |
|  | IPI00925621 |
|  | IPI00211004 |
|  | IPI00113879 |
|  | IPI00124823 |
|  | IPI00563608 |
|  | IPI00653610 |
|  | IPI00929886 |
|  | IPI00337688 |
|  | IPI00167680 |

---
